# Exploiting epigenetic dependencies in ovarian cancer therapy

**DOI:** 10.1002/ijc.33727

**Published:** 2021-08-06

**Authors:** Aisling Y. Coughlan, Giuseppe Testa

**Affiliations:** ^1^ Department of Experimental Oncology European Institute of Oncology, IRCCS Milan Italy; ^2^ Department of Oncology and Hemato‐oncology University of Milan Milan Italy

**Keywords:** chromatin remodeling, disease modeling, epigenetic drugs, ovarian cancer, precision oncology

## Abstract

Ovarian cancer therapy has remained fundamentally unchanged for 50 years, with surgery and chemotherapy still the frontline treatments. Typically asymptomatic until advanced stages, ovarian cancer is known as “the silent killer.” Consequently, it has one of the worst 5‐year survival rates, as low as 30%. The most frequent driver mutations are found in well‐defined tumor suppressors, such as *p53* and *BRCA1/2*. In recent years, it has become clear that, like the majority of other cancers, many epigenetic regulators are altered in ovarian cancer, including *EZH2*, *SMARCA2/4* and *ARID1A*. Disruption of epigenetic regulators often leads to loss of transcriptional control, aberrant cell fate trajectories and disruption of senescence, apoptotic and proliferation pathways. These mitotically inherited epigenetic alterations are particularly promising targets for therapy as they are largely reversible. Consequently, many drugs targeting chromatin modifiers and other epigenetic regulators are at various stages of clinical trials for other cancers. Understanding the mechanisms by which ovarian cancer‐specific epigenetic processes are disrupted in patients can allow for informed targeting of epigenetic pathways tailored for each patient. In recent years, there have been groundbreaking new advances in disease modeling through ovarian cancer organoids; these models, alongside single‐cell transcriptomic and epigenomic technologies, allow the elucidation of the epigenetic pathways deregulated in ovarian cancer. As a result, ovarian cancer therapy may finally be ready to advance to next‐generation treatments. Here, we review the major developments in ovarian cancer, including genetics, model systems and technologies available for their study and the implications of applying epigenetic therapies to ovarian cancer.

**Abbreviations**
ARID1A: AT‐rich interactive domain‐containing protein 1a
CARM1, aka PRMT4: coactivator‐associated arginine methyltransferase 1
CHD: chromodomain helicase DNA‐binding
HDAC: histone deacetylase
HGSOC: high‐grade serous ovarian cancer
ISWI: imitation switch
KDM: lysine demethylase
KMT: lysine methyltransferase complex
LGSOC: low‐grade serous ovarian cancer
OCCC: ovarian clear cell carcinomas
OEC: ovarian endometrioid carcinomas
PARP: poly (ADP‐Ribose) polymerase
PRC: Polycomb Repressive Complex
PRMT: protein arginine methyltransferase
PTMs: post‐translational modifications
SCCOHT: small cell carcinoma of the ovary hypercalcemic type
SWI/SNF, aka BAF: switch/sucrose non‐fermentable

## 1 INTRODUCTION

Ovarian cancer is one of the most lethal gynecological malignancies, with the highest incidence rates in North America, as well as Central and Eastern Europe. When diagnosed at Stage I, the 5‐year survival rate is ~90% for all subtypes. However, due to a lack of symptoms prior to metastasis throughout the abdomen, and the failure of current treatments to lead to complete remission, the vast majority of serous carcinomas are not diagnosed until Stage III (51%) or IV (29%).^1^, ^2^ Thus, the overall 5‐year survival rate is as low as 30% worldwide.^3^, ^4^, ^5^ The vast majority of ovarian tumors originate from one of three cell types: epithelial (>90%), stromal (~6%) and germ cells (2%‐3%).^3^, ^5^, ^6^

**FIGURE 1:**
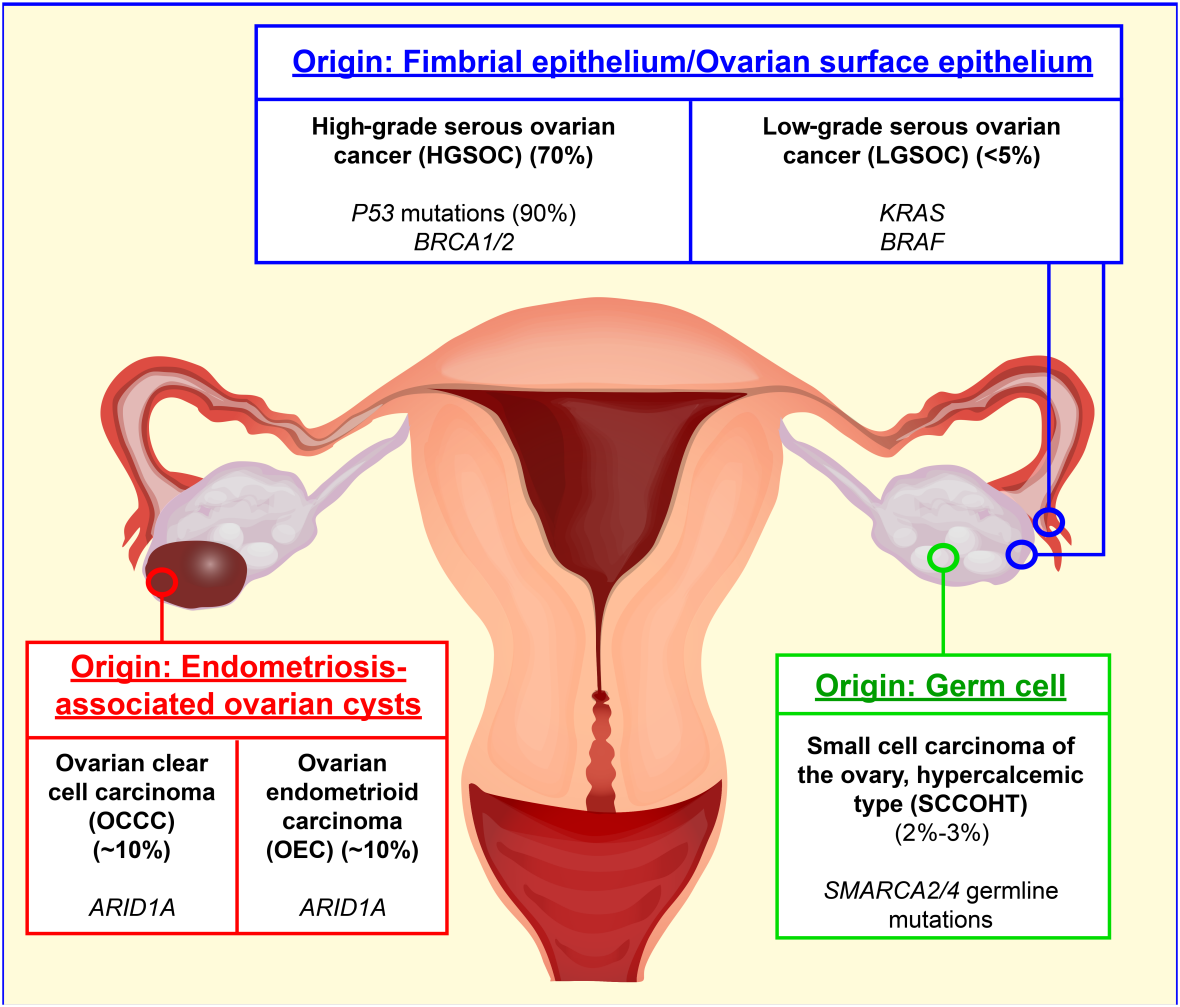
Current understanding of the putative tissues of origin for key ovarian cancer subtypes, and the most frequent mutations associated with each type

Each of these subtypes of ovarian cancer has distinct clinical features, putative cells of origin and associated driver mutations. This suggests different pathways driving the alternative cancer types, and specific weaknesses or synthetic lethalities should exist for each. As an example of inter‐subtype variation, high‐grade serous ovarian cancer (HGSOC) typically occurs in post‐menopausal women and is characterized by frequent (>90%) P53 mutations.^6^, ^7^, ^8^, ^9^ This is the most common form of ovarian cancer, accounting for 70% of all cases.^3^, ^5^, ^6^ A highly aggressive malignancy, its cell of origin is often unclear, there has been ambiguity as to whether it has originated from ovarian surface epithelium or tubal epithelium, with recent data suggesting that it can arise from both (Figure 1).^3^, ^10^ Emerging technologies in cell of origin tracing, such as OriPRINT,^10^ and patient‐derived organoids for normal tissue comparative analysis,^11^ we are closer to tracking the specific dysregulation driving tumorigenesis.

The key driver of this type of ovarian cancer, p53, is of course a common genetic lesion in many cancers, which causes genomic instability and structural variation; however, the p53 pathway has proven to be difficult to target therapeutically.^12^ For these reasons, this review will focus on alternative genetic and epigenetic disruptions in the different subtypes of ovarian cancer, such as amplification and hyperactivity that may represent more viable targets for cancer therapy.

A related subtype, low‐grade serous ovarian cancer (LGSOC) typically presents in patients between 40 and 55 years of age and is associated with mutations in *KRAS* and *BRAF*.^4^, ^13^ On the other hand, ovarian clear cell carcinomas (OCCC) and ovarian endometrioid carcinomas (OEC) frequently feature mutations in *PTEN* and *ARID1A*.^3^, ^4^ OCCC and OEC are the second and third most common histological subtypes, respectively, that together account for ~20%‐25% of epithelial ovarian cancer cases. These cancers are thought to originate through endometriosis and endometriotic ovarian cysts (Figure 1). Their specific cell of origin remains unclear but related to ectopic inclusions that are suspected to be tubal or endometrial in origin.^3^, ^14^, ^15^, ^16^

The final subtype we will discuss is small cell carcinoma of the ovary. This is a very rare and highly aggressive cancer that typically occurs at a young age when compared to other ovarian cancers (~23.9 years mean).^17^ Small cell carcinoma of the ovary hypercalcemic type (SCCOHT) is almost exclusively defined by germline or somatic mutations in SMARCA4, the ATPase subunit of the BAF complex, and is currently thought to originate from germ cells (Figure 1).^18^, ^19^, ^20^

Currently, standard treatment for newly diagnosed ovarian cancer is a combination of surgical cytoreduction and platinum‐based chemotherapy.^21^ At advanced stages, recurrence and platinum‐resistance of tumors is very common.^4^, ^22^ In recent years however, there has been significant advancement in the use of directed therapies, such as inhibition of angiogenesis and DNA repair pathways.^23^, ^24^, ^25^ Inhibition of the DNA repair enzyme poly (ADP‐Ribose) polymerase (PARP) has shown specific activity in HGSOC with homologous recombination deficiency (~50%), with particular efficacy in *BRCA*‐mutated HGSOC (~20%).^8^, ^21^, ^24^, ^26^ Despite such promising advances in directed therapy, 50%‐80% of HGSOC patients do not contain the sensitive mutational profile to benefit from these therapies. This highlights the urgent need for additional therapeutics for this malignancy, targeting alternative mutational sensitivities.

It has become clear in recent years that disruption of the epigenetic machinery is key in development in almost all human cancers.^27^ This discovery has unlocked vast potential for the field of oncology, as the plasticity of chromatin states makes epigenetic machinery attractive targets for cancer therapy. Epigenetics—the reversible modification of both DNA and DNA‐bound histones^28^—regulate the underlying genes, and disruption of the machinery results in aberrant activation/repression of key cancer‐related genes, driving oncogenesis. Therapies targeting oncogenic deregulation of the epigenome, such as EZH2 and BET‐family inhibition, have been successfully used in clinical and preclinical trials for many diverse cancers, such as non‐Hodgkins lymphoma and glioblastoma.^27^ This review will focus on the key perturbations to epigenetic machinery in ovarian cancer, the sensitivities these disruptions create that can be exploited by available therapies and the advances in technologies that are bringing us closer to precision oncology for ovarian cancer.

## 2 EPIGENOMIC REGULATION OF TRANSCRIPTION

Over the past century, it has become clear that the organization of DNA in 3D space is one of the most fundamental controls of gene expression. Genetic information must be, in the simplest sense, physically accessible in order to be regulated. Accessibility is determined by position and information loaded in the histone‐DNA complex known as the nucleosome. The nucleosome is comprised of 147 base pairs of DNA wrapped around an octamer of histones H2A, H2B, H3 and H4.^29^, ^30^ Nucleosome remodeling is the alteration of the histone‐DNA interface by a dedicated set of chromatin remodeling enzymes. There is a large and complex interplay between remodelers responsible for positive and negative regulation of genetic regulatory elements. Four main subfamilies of chromatin remodelers exist, based on domain structure and organization of the catalytic ATPase translocase domain; chromodomain helicase DNA‐binding (CHD), INO80, imitation switch (ISWI) and switch/sucrose non‐fermentable (SWI/SNF, aka BAF) are the main subgroups, each of which can have distinct targets activities based on associated accessory proteins. BAF alters chromatin access through nucleosome repositioning/eviction, whereas members of the INO80 family can edit the composition of nucleosomes through incorporation of histone variants.^30^, ^31^ Fundamentally, chromatin remodeling enzymes (remodelers) control transcription through mobilization and organization of nucleosomes to make genetic information more or less accessible to transcriptional machinery.^31^

The octamer of histones comprising the nucleosome contains post‐translational modifications (PTMs) with specific information on how the associated DNA should be organized. PTMs to the N‐ and C‐terminal tails of histones are added and removed by enzymes commonly referred to as “writers” and “erasers,” ushering in both physical changes (compaction/relaxation) and/or relay signals to be executed upon the associated regions of DNA via the actions of effector proteins (“readers”). The compaction state of a DNA region—influenced by its histone PTM status—is the central control to transcriptional output, either allowing or restricting its access to downstream transcription factors.^32^, ^33^ Some of the most common histone modifications include methylation, acetylation, ubiquitination and phosphorylation. Designated enzymes catalyze the transfer (writers) or removal (erasers) of these marks to specific amino acid residues. Regulators (readers) recognize these marks, and through this recognition recruit other regulators, enzymes or remodelers conferring downstream changes in chromatin compaction and accessibility.^32^, ^33^

For example, mono‐, di‐, or trimethylation of histone lysines is catalyzed by six major groups of lysine methyltransferase complexes (KMT1‐6), the lysine methyl “writers.” Key examples are EZH1 and EZH2 of the KMT6 family, which are the catalytic subunits of Polycomb Repressive Complex (PRC) 2, designating H3K27 mono‐, di‐ and trimethylation.^34^, ^35^, ^36^ These “writing” enzymes catalyze the transfer of methyl groups to specific lysine residues, but the plasticity of chromatin requires the changing of states during development, transitioning from pluripotent transcriptional profiles to differentiated expression profiles through dynamic writing and erasing of key genomic sites. Thus, several families of “erasers” counteract the activity of the writer enzymes. Key examples are histone lysine demethylases (KDM) such as UTX, which removes methyl groups from H3K27.^37^, ^38^

The balanced writing and erasing modification states ultimately serves as information understood and acted upon by “reader” enzymes. For instance, H3K27me3 is specifically recognized and bound by the Cbx components of the PRC1 complex and is involved in chromatin compaction.^39^, ^40^ Thus, the complex interplay and balance of these writers, erasers and readers is key to transcriptional regulation and cellular identity.

As chromatin modification and remodeling has such a critical role in transcription, it is unsurprising that mutations in fundamental transcriptional regulators are commonly disrupted in cancers. Genomic analysis of various cancers indicates that chromatin remodelers are some of the most frequently mutated genes in human malignancies.^31^, ^41^ Of specific interest are the antagonistic relationships between BAF and Polycomb complexes, as well as the arginine methyltransferase CARM1. Here we discuss some of the most important chromatin regulatory complexes, their role in ovarian cancer and the therapeutic opportunities these lesions create.

## 3 POLYCOMB REPRESSIVE COMPLEXES 1 AND 2

Polycomb group proteins are highly conserved chromatin repressors responsible for cell fate gene regulation during development and play a fundamental role in cellular identity. Mammals contain two main multi‐subunit PRC, PRC1 and PRC2.^34^ PRC1 catalyzes the ubiquitination of H2AK119ub1, a modification essential for repression at Polycomb target promoters.^42^, ^43^ It can be broadly divided into two subcomplexes (canonical and variant) that contribute to repression in alternative fashions. vPRC1 (variant) catalyzes the majority of H2AK119ub1, which promotes repression at promoters, at least partially, through PRC2 recruitment,^42^, ^44^ while cPRC1 (canonical) promotes compaction and long‐range repressive Polycomb body formation through subunits that confer structural changes on chromatin.^40^, ^45^, ^46^

PRC2 is responsible for mono‐, di‐ and tri‐methylation of H3K27. Tri‐methylation is typically found at repressed promoters, where it is essential for maintaining transcriptional repression of non‐lineage expressed genes. Di‐methylation is found across the genome, where it has been suggested to maintain repression of tissue‐specific enhancers^47^ while mono‐methylation is found at active gene bodies, though the function, if any, of this has not yet been established. The PRC2 core contains three subunits EED, SUZ12 and a catalytic methyltransferase component, either EZH1 or EZH2.^48^ It has become clear that mutations in Polycomb complexes and consequent aberrant H3K27 methylation patterns have a key role in cancer development.^34^ In these cases, failure to undergo cellular differentiation is a consequence of aberrant methylation patterns of H3K27.^49^, ^50^

### 3.1 EZH2

EZH2 is widely mutated in various cancer types and can act in both an oncogenic or tumor suppressive capacity.^34^ “Change of function” mutations are observed in diffuse large B‐cell and follicular lymphomas, and to a lesser degree in melanoma, which cause global hyper tri‐methylation of H3K27, replacing the diffuse intergenic H3K27me2 levels.^34^, ^51^ Conversely, EZH2, SUZ12 and EED deletions, which cause reductions in H3K27me3 levels, are also observed in leukemias and other myeloid disorders.^52^, ^53^ This is an example of the importance of mutational context in cancer development and reinforces the need for patient‐specific profiling in order to choose appropriate therapeutics, as will be discussed later in this review.

**FIGURE 2:**
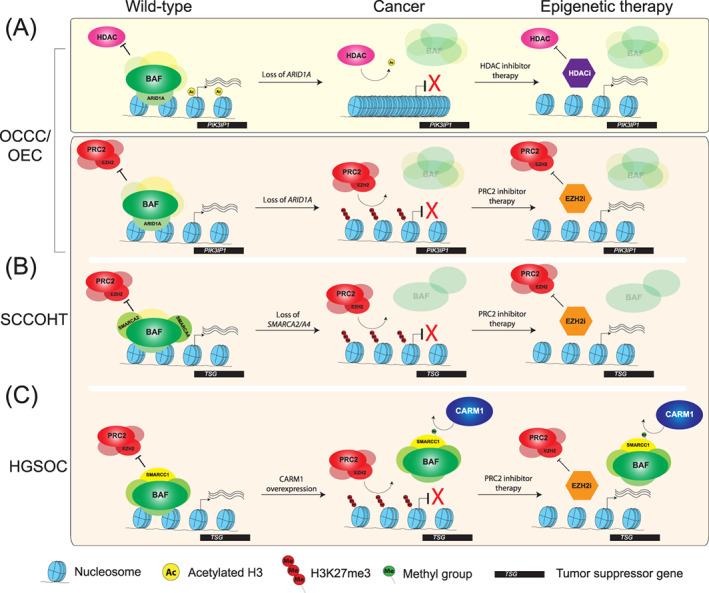
Key regulatory mechanisms in OCCC/OEC (A), SCCOHT (B), and HGSOC (C), highlighting the consequences of their disruption, the molecular sensitivities created, and how these perturbations can be exploited for therapy by epigenetic inhibitors

EZH2 overexpression/amplification has been widely reported in ovarian cancers. Increased EZH2 activity has been found in ~85% of epithelial ovarian carcinomas through genetic amplification and loss of antagonistic protein activity^54^, ^55^, ^56^, ^57^ and is functionally implicated in tumor development and proliferation.^56^, ^58^ While studies show in vitro cytotoxic effects of EZH2 inhibition against several ovarian cancer lines,^58^ the efficacy of EZH2 targeting is highly dependent on the mutational status of several other key epigenetic regulators, which can be cooperative or antagonistic to EZH2 function, rather than the mutational status of EZH2 itself (Figure 2).^55^, ^59^, ^60^, ^61^, ^62^, ^63^ There is a balanced and specific interplay between chromatin regulators to maintain cellular identity and accurate gene expression. Loss of antagonists to PRC2, such as BAF components ARID1A and SMARCA2/4, leads to unrestricted EZH2 activity and improper repression of key tumor suppressor genes. This has been shown to be a fundamental driver in multiple subtypes of ovarian cancer.^64^, ^65^, ^66^, ^67^ To understand the action and potential of EZH2 inhibition in ovarian cancer, we must first discuss the mechanism of action of these PRC2 antagonists whose loss results in EZH2 dependency in cancer.

## 4 BAF

One of the best‐known nucleosome‐remodeling complexes is BAF. Its catalytic activity is important for accessibility and activation of genes, binding active promoters and enhancers through recognition and recruitment to H3K4me1 and multiple bromodomain‐containing subunits reading histone acetylation.^31^ Originally identified in yeast as a major transcriptional regulating complex, it has since been found that human BAF comprises multiple configurations and compositions.^68^ It contains a core set of essential components, the BAF‐core. Combinations of subunits build upon this to define three distinct compositions—BAF, PBAF and ncBAF.^31^, ^69^ ARID1A/B and DPF1/2/3 specify the BAF complex. PBRM1, BRD7, ARID2 and PHF10 define the PBAF complex, and ncBAF‐specific components include BRD9 and GLTSCR1/1L.^31^ The presence of distinct accessory proteins specializes the complexes, directing them to primarily non‐redundant sets of genomic targets for each of these complexes, such as recruitment to active sites through the multiple acetyl lysine readers.^70^

A large body of evidence has accumulated implicating a role for the BAF complex in tumor suppression.^41^, ^71^ In fact, components of BAF machinery are mutated in ~20% of human cancers, making it the most frequently mutated chromatin regulatory complex across human malignancies.^41^ Consequently, identifying therapeutic susceptibilities of cancers carrying BAF mutations could be broadly applicable, particularly as understanding the mechanisms of specific BAF complex mutations has led to the discovery of targetable synthetic lethalities.^31^, ^72^, ^73^, ^74^, ^75^ Indeed, the BAF components ARID1A and SMARCA2/4 are persistently mutated in ovarian cancer subtypes,^64^, ^65^, ^66^, ^67^ and represent promising sensitivities for epigenetic inhibition therapy, as discussed below.

### 4.1 ARID1A

AT‐rich interactive domain‐containing protein 1a (ARID1A) is a component of the BAF complex involved in the regulation of multiple genes. ARID1A is the most frequently mutated subunit of the BAF complex across all human malignancies.^69^ ARID1A is mutated in ~57% of OCCC and ~30% of OEC, with the majority being frameshifts and nonsense, likely loss‐of‐function, mutations.^59^, ^64^, ^66^, ^76^, ^77^ Its null mutations result in altered expression of several key genes such as *CDKN1A* and *PIK3IP1*,^59^, ^65^, ^66^ both of which regulate entry into apoptosis and are aberrantly repressed in the absence of ARID1A due to changes in chromatin remodeling.^66^ Mouse models with a single mutation in *PIK3CA* (a frequently co‐occurring mutation with ARID1A) have been shown to rapidly develop OCCC‐like tumors upon *ARID1A* loss.^78^

The established antagonistic relationship between the Polycomb complexes and the BAF complex logically predicts that loss of function of BAF components will lead to unopposed activity of repressive Polycomb machinery.^31^, ^79^ Thus, repression of key apoptotic‐entry proteins in tumor cells can be expected to rely largely on EZH2 activity and suggests Polycomb complex components as promising therapeutic targets for the majority of OCCC and a significant proportion of OEC.^55^, ^74^, ^80^ Indeed, *ARID1A*‐mutant ovarian cancer cell lines OVISE and TOV21G have displayed sensitivity to EZH2 inhibition, and in wild‐type ARID1A cells, its knockdown confers EZH2 sensitivity.^55^, ^59^ Without ARID1A, unrestrained activity of EZH2 represses ARID1A target genes, such as *PIK3IP1*. Once EZH2 is inhibited, PIK3IP1 repression is relieved, leading to apoptotic cell death in tumor cells (Figure 2A). Importantly, in wild‐type *ARID1A* cells EZH2 inhibition did not display any significant impact on cell proliferation demonstrating the specificity of this effect to ARID1A mutant ovarian cancers and underscoring the critical actionability of patients' tumor genotype in clinical practice,^59^ and more specifically the way in which patient‐specific mutations and a mechanistic understanding of dysregulation in chromatin pathways can jointly inform rational therapeutic innovation.

Another prospective therapeutic target in ARID1A mutant cancers is histone deacetylase (HDAC) activity. Antagonism between the BAF complex and HDAC containing complexes such as NuRD is critical in maintaining chromatin state and cellular identity, and failure to maintain this balance contributes to cancer development.^31^, ^81^ In the absence of functional ARID1A, the overactivity of multiple HDACs results in aberrant repression of pro‐apoptotic genes, including p53 and *PIK3IP1*.^82^, ^83^ It has been reported that *ARID1A* mutant cells become dependent on HDAC6 activity, as HDAC inhibition results in accumulation of acetylated p53 Lys‐120—a pro‐apoptotic modification (Figure 2A).^82^ In *ARID1A* mutant cells, PIK3IP1 is repressed through cooperative activity of EZH2 and HDAC2, and upon treatment with HDAC inhibitors, PIK3IP1 is derepressed and promotes cell death. Mouse models of OCCC lacking ARID1A activity display increased survival and reduced ascites accumulation and tumor progression when treated with the pan‐HDAC inhibitor SAHA,^83^ making HDAC inhibition a very promising strategy for mutant *ARID1A* OCCC and OEC. In fact, the HDAC6 inhibitor ACY‐1215 is already in clinical trials for lymphoma, multiple myeloma and breast cancer (https://clinicaltrials.gov/) and may potentially be repurposed for OCCC or OEC. Growing evidence suggests a synergistic anti‐cancer action of the combination of EZH2 and HDAC inhibition in several cancers.^84^, ^85^ There is an increasing number of clinical trials implementing precision medicine and biomarker‐directed therapy, as well as combination treatments epigenetic drugs and established anti‐cancer therapies.^86^ Given the evidence for epigenetic intervention as a strategy for ovarian cancer, this could be a very promising approach in the clinical setting.

### 4.2 SMARCA2/4

The human BAF complex has two separate ATPases, SMARCA2 and SMARCA4 (also known as BRM and BRG1, respectively). Each individually can combine with approximately eight other core subunits to form the BAF core, which in turn assemble into BAF, PBAF or ncBAF. SMARCA2/4 act as the molecular motor for the complex, using ATP hydrolysis to power the movement of the complex over DNA resulting in the local repositioning of nucleosomes. Mutations in SMARCA4 occur in a wide range of cancers, including lung cancers,^87^ melanomas^88^ and lymphomas.^89^ Critically, almost every case of SCCOHT is characterized by inactivating mutations in *SMARCA2* and/or *SMARCA4*.^67^, ^74^, ^90^, ^91^

A promising clinical development has also been the sensitivity of *SMARCA4* null cells to *EZH2* inhibition (Figure 2B). SCCOHT cell lines display synthetic lethality with core PRC2 complex subunits, which is supported by experiments showing that SCCOHT cells are acutely sensitive to EZH2 inhibition, inducing cell cycle arrest and apoptosis.^60^, ^61^ Considering the frequency of SMARCA2/4 loss in SCCOHT, EZH2 inhibition, or other PRC2 inhibitors (EZH1/2 dual or EEDi), would appear to be an excellent and highly effective choice, as has been suggested in several studies.^74^ However, what has been overlooked is that cases of SCCOHT are frequently familial, predisposed by germline mutations in *SMARCA4*.^67^ While EZH2 inhibitors are tolerated in *SMARCA4* wild‐type patients in clinical trials to date, patients with germline *SMARCA4* mutations instead may be inherently sensitive to EZH2 inhibition. To date, studies have been carried out using cell lines with homozygous deletions of *SMARCA4*, displaying sensitivity to EZH2,^60^ or xenografting *SMARCA4+/+* mice with cell lines bi‐allelic for loss of *SMARCA4*.^59^, ^92^ A key experiment that, to our knowledge, has not yet been carried out would be testing the toxicity of EZH2 inhibition on SMARCA4 heterozygous cell lines and mice where side‐effects compared to previous trials may be greater. In addition, the capacity to generate iPSCs from patients with *SMARCA4* germline mutations could be a critical paradigm for defining patient‐specific efficacy and toxicity of treatments, well beyond the predictive capability of the mouse model.

## 5 CARM1

Protein arginine methyltransferases (PRMTs) catalyze the methylation of arginine side chains, a fundamental process in the regulation of mRNA splicing, signal transduction, DNA repair and gene expression.^93^, ^94^ In addition, arginine methylation modulates the activity of many cancer‐associated proteins.^94^, ^95^ Coactivator‐associated arginine methyltransferase 1 (CARM1, also known as PRMT4) is an arginine methyltransferase essential during mammalian development.^96^ It catalyzes asymmetric dimethylation of arginines on a small set of distinct substrates, including histone 3.^97^ Its recruitment to promoters results in increased levels of H3R17 and H3R26 methylation, which are associated with active transcription.

CARM1 is overexpressed in multiple cancer types, including breast,^98^, ^99^ colorectal^100^ and HGSOC.^62^, ^94^, ^101^ It is involved in the activation of several cancer‐related genes, including cyclins and beta‐catenin.^94^, ^98^, ^100^ Other than its histone substrates, CARM1 also methylates substrates involved in epigenetic chromatin remodeling such as SMARCC1 (BAF155). CARM1 forms a complex with ATP‐remodeling (BAF) factors, and its methylation of SMARCC1 results in the eviction of the BAF complex from target loci (Figure 2C).^99^ The precise mechanism of how this methylation redirects BAF targeting is not entirely clear. However, EZH2 and SMARCC1 antagonistically regulate several key tumor suppressors, such as the apoptosis‐promoting gene *NOXA*.^62^ Therefore, akin to ARID1A‐mutant OCCC, CARM1 overexpression and its consequent loss of SMARCC1 activity impairs BAF's counteraction of Polycomb repression, leading to replacement by EZH2 (Figure 2C).^62^, ^63^, ^99^ Indeed, EZH2 inhibition is selectively effective against CARM1‐overexpressing HGSOC,^62^, ^63^ and thus represents a promising therapeutic strategy in this setting as well (Figure 2C).

## 6 BROMODOMAIN PROTEINS

Multiple bromodomain‐containing proteins may be effective targets in ovarian cancer. One such bromodomain protein, BRD4, is a chromatin reader protein crucial during embryogenesis and cell differentiation. It, like other bromodomain‐containing proteins, binds acetylated histones at transcription start sites and active enhancers, recruiting transcriptional machinery to chromatin.^102^, ^103^, ^104^, ^105^ Many cancerous cells aberrantly hyperactivate oncogenes, such as *MYC*, through co‐option of non‐native enhancers and BRD4 activity.^106^ BRD4 inhibition therefore may be selectively toxic to cancers dependent on such enhancer or super‐enhancer activity. BRD4‐occupied enhancers function within phase separated punctae, within which drugs such as cisplatin accumulate and show specific and greater activity. The manipulation or disturbance of such particles may therefore greatly impact the efficacy of current cancer therapies.^107^ In fact, recent evidence implicates disruption of condensate‐forming mechanisms by cancer cells as a mechanism of chemo‐resistance,^107^ one of the major issues in ovarian cancer long‐term therapy.^22^ Therefore, the nature of BRD4 activity makes it a promising broad spectrum target, especially in the instance of chemo‐resistance to cisplatin.

The expression of BRD4 target genes is frequently altered in oncogenesis.^102^, ^103^ Its overexpression and consequent oncogenic transcriptional profiles have been reported in HGSOC and are associated with poor overall survival.^108^, ^109^ However, the enhancer landscape of ovarian carcinomas remains largely unexplored. Multiple inhibitors have been developed for targeting BRD4, which are already in clinical and pre‐clinical trials^27^, ^110^ that may be effectively applied to HGSOC. Indeed, HGSOCs displaying increased BRD4 expression and its transcription profile exhibit sensitivity to BET inhibition.^111^ Considering the current poor survival rates and intractability of HGSOC, it is promising that there may already be multiple avenues of effective epigenetic treatment approved and available for repurposing.

Another bromodomain‐containing protein of significance to ovarian cancer is BRD9. Implicated in oncogenic profiles of several cancers, it is a key component of the GLTSCR1‐BAF (ncBAF or GBAF), a remodeling complex targeting multiple loci that are involved in pluripotency.^112^ Perturbations in canonical BAF components have been shown to cause a dependence on ncBAF activity^75^ and BRD9 inhibition with a small‐molecule degrader successfully reverses of the oncogenic transcription pattern and significantly impedes cell proliferation in cancers associated with perturbations in the BAF‐core.^73^, ^75^ Due to the frequent mutation of BAF components in ovarian cancer discussed here, targeting key ncBAF components may be of great potential. A recent study demonstrated that BRD9 levels are in fact elevated in ovarian cancer, and that inhibition sensitizes ovarian cancer to PARP inhibition and cisplatin therapy through regulation of the DNA damage response machinery—a frequently disrupted pathway in ovarian cancer.^113^, ^114^ The success of the BRD9 degrader in clinical trials will be of great significance for future ovarian cancer therapy.

## 7 FUTURE STEPS FOR THERAPEUTIC ADVANCES

**FIGURE 3:**
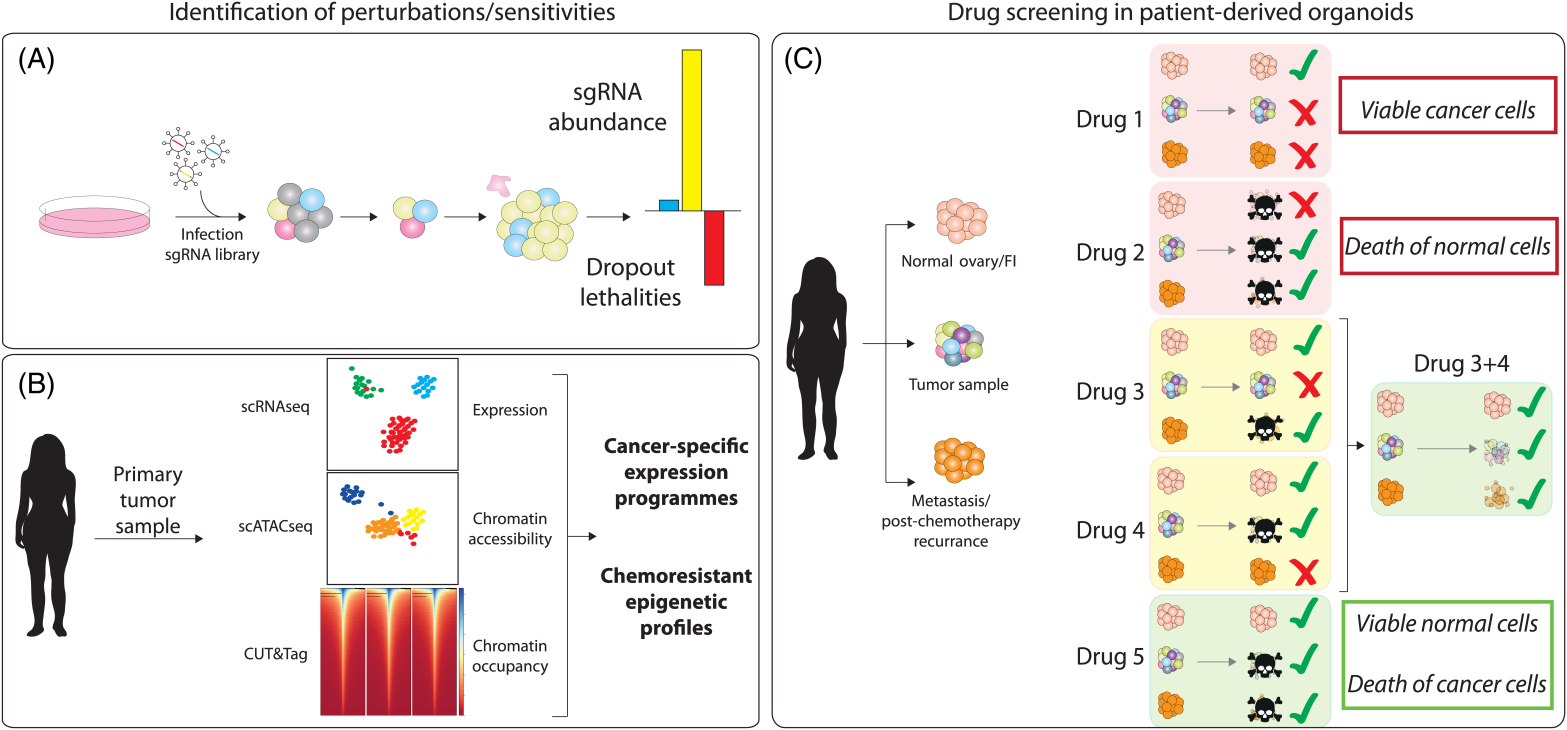
Emerging technologies for identifying improved patient‐specific treatments, illustrations of (A) CRISPR screening for identification of synthetic lethalities, (B) single‐cell technologies to identify cancer‐specific epigenetic/transcriptional changes and (C) patient‐specific organoid modeling as a basis for personalized drug screening and therapeutic discovery

What is clear from the evidence above is that the efficacy of EZH2 inhibition in multiple ovarian cancers (and indeed many other malignancies) is dependent on the mutational context, copy number or activity of other genes, that is, ARID1A, SMARCA4 and CARM1 rather than on Polycomb complex components themselves. This clearly indicates the importance of identifying individual patient mutational profiles for effective and directed therapy. Broad‐spectrum cancer therapies have limited utility, even within cancer subtypes. Ovarian cancer itself is subdivided into distinct categories, based on cell of origin, histology and pathogenesis. But, as discussed above, within these categories the underlying mutational and epigenetic landscapes vary greatly, and treatment that may be successful in one individual may be ineffective or even potentially harmful to another. In short, the key step forward in ovarian cancer treatment, and indeed cancer treatment in general, is to profile and define the specific mutational background of cells within individual tumors. Additionally, fully characterizing the cell‐of‐origin of the various subtypes, through techniques such as OriPRINT,^10^ will allow proper molecular characterization and identification of subtype‐specific vulnerabilities. The burgeoning fields of organoid modeling, single‐cell technologies and epigenetic characterization can guide us through this complex area (Figure 3).

### 7.1 Organoid modeling

Organoids mimic the fundamental characteristics of organs as multicellular, 3D in vitro cultures, derived from primary cells.^115^, ^116^ Originally, it was revealed that single intestinal stem cells could give rise to self‐organizing differentiated structures of the gut when provided with key factors for the in vivo intestinal stem cell niche.^115^, ^116^ With this paradigm, through modification of the cocktail of factors to the specific organ, there are now organoids developed that faithfully recapitulate key features of multiple vital organs, including breast, kidney and stomach.^117^, ^118^, ^119^

Critically, 3D tumor organoids have been successfully developed for many malignancies.^116^, ^120^, ^121^ They are already demonstrating promising results in preclinical prediction of treatment response to both targeted therapies and chemotherapeutics,^122^ to improve and inform therapeutic options for broadly treatable tumors^123^ and also as models to understand and characterize the mechanism of pathogenesis of intractable malignancies, such as pancreatic ductal adenocarcinoma.^124^

Patient‐derived organoid models have recently been developed for ovarian cancer, through several approaches that recapitulate the genomic landscape and tumor heterogeneity of primary ovarian cancer tumors.^11^, ^125^ One of the key challenges in ovarian cancer is the capacity of a small subpopulation of quiescent cells to evade treatment, and results in post‐chemotherapy relapse.^126^ It is becoming clear that a combination of therapies targeting individual susceptibilities of cancer subpopulations may lead to greater disease‐free survival rates. In combination with single‐cell omics technologies, organoids represent a transformative approach for making tumor heterogeneity experimentally tractable in vitro and profiling patients as to the mutational susceptibilities of their tumors (Figure 3C).

In addition, and perhaps most critically, organoid technology can be used for comparative analysis of potential treatments in tumor vs normal tissues (Figure 3C). For example, if we consider the case of the variable success of EZH2 inhibitors, characterizing *ARID1A* and *SMARCA4* mutational status will provide valuable data as to the likely efficacy of EZH2, as mutants in both of these genes display dependency on EZH2 activity.^55^, ^59^, ^60^, ^61^ However, the question of whether the mutation is germline or somatic remains to be answered, with particular reference to germline SCCOHT *SMARCA4* mutations. The burgeoning field of organoid modeling could be extremely beneficial to tackle this question. We can utilize patient‐derived normal and cancer organoids to comparatively assess the relative cytotoxicity of inhibitors to normal tissues that are specific to the patient, not just to the tumor itself.

### 7.2 Chromatin profiling and single‐cell technologies—deconvoluting tumor heterogeneity

The application of single‐cell transcriptomics to cancer has been illuminating in the deconvolution of tumor heterogeneity and has already been successful in identifying druggable pathways^127^ and understanding sub‐populations resistant to drug treatment (Figure 3B).^128^ Analysis of tumor cell populations pre‐ and post‐chemotherapy could be a fundamental in improving long‐term survival in ovarian cancer patients.

Disruption of chromatin remodeling is clearly a fundamental property in ovarian cancer. Understanding the impact of such mutations on activity and chromatin accessibility in cell lines can be readily profiled using bulk techniques, such as standard ChIP and ATAC‐seq. This is particularly relevant for BAF mutant cancers as they are a key regulator of accessibility at enhancers and promoters.^74^ However, for organoid technology and patient‐specific profiling, obtaining the required cell number is a major hindrance. Primary samples have limited utility due to their propensity to undergo senescence, limiting the expansion capability and reducing input. The application of new technologies with reduced requirements for cell input overcomes such issues, such as CUT&Tag—an alternative approach to ChIP for detection of chromatin‐bound proteins using a tethered Tn5 transposase fusion protein. An antibody to the protein of interest is recognized and bound by the Tn5, which then specifically tagments adjacent DNA.^129^ This technology reduces the required cell number from millions (standard ChIP‐seq) to several thousands, as well as allowing single‐cell applications^130^ with the potential to increase resolution, throughput and elucidate mechanisms previously masked through bulk epigenomic analyses.

### 7.3 CRISPR screening

CRISPR‐based screening has been successfully used to identify synthetic lethalities in multiple cancers.^73^, ^131^ Identified hits often translate to logical sensitive targets to inhibition or degradation therapies. Applying this paradigm in ovarian cancer cell lines and organoids is a major step toward identifying druggable sensitivities (Figure 3A). Further classification of the intra‐tumor cell subpopulations, and the combination therapies most effective for complete cancer clearance, relies on integrative screening of organoids, CRISPR/Cas perturbation technology and single‐cell applications to fully understand the process of ovarian cancer tumorigenesis and optimize therapeutics screening and strategies (Figure 3).

The ever‐improving technologies in the field of CRISPR screening, such as knockout, activation, Perturb‐seq and domain tiling screens, have rapidly increased our ability to probe specific weaknesses in cancer cells.^131^, ^132^, ^133^ Databases such as Depmap allow for simple identification of synthetic lethalities providing new and druggable options for targeting.^134^ Focused use of such screening technologies in ovarian cancer cell models such as 2D cell lines and organoids will enhance drug target identification in a patient and mutation specific context. The coming decade will see these technologies cumulatively harnessed in order to advance ovarian cancer treatment.

## 8 CONCLUSION

Ovarian cancer therapy and survival has not progressed significantly in over 30 years. Genetic and epigenetic variability within different cells in a tumor presents obvious difficulties to cancer treatment. It has become clear that therapies targeting tumors as a homogenous group of cells commonly result in drug‐resistant relapse.^135^ However, with the advent of new technologies, it is becoming more evident that many of the most common ovarian cancer subtypes, such as HGSOC and OCCC, have mutational profiles that could be targeted with therapies already in clinical and preclinical trials for other malignancies. In fact, with the recent approval of the EZH2 inhibitor Tazemetostat by the FDA for treatment of follicular lymphoma,^136^ improved therapy for ovarian cancer may soon be readily available. It is extremely promising that inhibitors already in use for clinical trials exhibit anti‐tumor effects for ovarian cancer. EZH2 inhibition is one of the most promising therapies discussed here for CARM1‐overexpressing HGSOC, *ARID1A*‐mutant OCCC/OEC and *SMARCA2/4*‐deficient SCCOHT. What is lacking is a more thorough genetic and molecular understanding at the patient‐specific level. Combining emerging technologies such as patient‐specific organoids, CRISPR/Cas targeting of oncogenic drivers and single‐cell technologies, we can begin to identify novel appropriate and effective therapies to eradicate the entire population of cells that comprise individual patients' cancer. We may be on the cusp of a new era of ovarian cancer therapy, as we may already have the tools at our disposal to investigate drug repositioning and combination therapies.

## CONFLICT OF INTEREST

The authors declare no conflicts of interest.
